# Scattered Ultrasonic Guided Waves Characterized by Wave Damage Interaction Coefficients: Numerical and Experimental Investigations

**DOI:** 10.3390/s22176403

**Published:** 2022-08-25

**Authors:** Christoph Humer, Simon Höll, Christoph Kralovec, Martin Schagerl

**Affiliations:** Institute of Structural Lightweight Design, Johannes Kepler University Linz, Altenberger Str. 69, 4040 Linz, Austria

**Keywords:** ultrasonic guided waves, structural health monitoring, wave damage interaction coefficients, non-reflective boundaries, piezoelectric wafer active sensors, scanning laser Doppler vibrometer

## Abstract

The present paper comprehensively investigates the complex interaction between ultrasonic guided waves and local structural discontinuities, such as damages, through highly sensitive features: so-called wave damage interaction coefficients (WDICs). These WDICs are unique for each structural discontinuity and depend solely on their characteristics for a given structure and condition. Thus, they can be particularly useful for advanced assessment of lightweight structures in the context of non-destructive evaluation and structural health monitoring. However, the practical application of WDICs entails significant difficulties due to their sensitivity and complex patterns. Therefore, this study attempts to guide researchers and practitioners in the estimation of WDICs from numerical simulations and physical experiments. Detailed investigations are made for an aluminum host plate modified by artificial structural discontinuities, i.e., surface-bonded steel sheets. The numerical simulations are performed to predict WDICs and study sensitivities using a sophisticated finite element model. The experimental setup uses piezoelectric transducers to excite guided waves in the host plate. A single scanning laser Doppler vibrometer measures the scattered guided waves caused by the surface-bonded steel sheets, and the resulting WDICs with possible influences are investigated. In both cases, the orientation and thickness of the attached steel sheets were varied to create 12 different damage scenarios. In general, the comparison between numerical and experimental WDICs show good agreement. This underpins the applicability of the general methodology for simulating and measuring WDICs over all scenarios. Furthermore, the WDIC scattering patterns reveal a clear dependency of the peaks in the back-scattered reflections for both the numerical and experimental amplitude coefficients on the damage orientation, basically following the law of reflection. However, some discrepancies between both studies were observed. Numerical sensitivity analysis identified the adhesive layer as one reason for such differences. Additionally, misalignment errors in the experimental measurements were also found to affect WDICs. Therefore, an improved baseline subtraction method is proposed, which clearly enhances the experimental WDICs. Finally, an experimental sensitivity study of WDICs for selected sensing radii revealed only a minor influence. All these investigations were made for the amplitude as well as the phase representation of WDICs. Thus, these findings may open the way to future research and development of techniques employing WDICs for advanced applications of non-destructive evaluation and structural health monitoring.

## 1. Introduction

Nowadays, increasing economic and environmental demands call for improved design of structures, e.g., aircraft, cars, wind turbines, etc., using optimized lightweight solutions. Generally, such lightweight structures have reduced safety margins for catastrophic failures due to damages caused by unlikely loads, e.g., environmental influences or misuse. Therefore, over the last decades, several non-destructive evaluation (NDE) methods have been developed to address this safety risk and detect damages before they reach a critical point [1]. Some of these methods can be adapted by integrating actuators and sensors, e.g., piezoelectric transducers, in the structure to provide it with self-sensing capabilities. Such so-called smart structures enable embedded ultrasonic NDE and structural health monitoring (SHM), the continuous on-board monitoring of the damage state of critical structural components [2].

Giurgiutiu [3] used so-called piezoelectric wafer active sensors (PWASs) for different dynamic methods evaluating the damage state of a structure. These small and inexpensive transducers can be easily attached to or even integrated into structures. Due to the reversible piezoelectric effect, these PWASs can be used as actuators and/or as sensors simultaneously. Hence, they can be used solely as sensor in passive methods, e.g., acoustic emission [4], or as both actuator and sensor in active methods, e.g., guided wave methods [5] and the electromechanical impedance method [6]. Guided waves can travel long distances trough thin-walled structures while interacting with structural changes such as damages, even those of small size. Hence, these ultrasonic waves provide the possibility to examine a large area from a single probing position and, thus, have stimulated research on their application in recent decades [7,8]. Generally, different levels for SHM tasks can be defined, i.e., detection, localization, quantification, and typification [9]. Ultrasonic guided wave-based methods seem promising to potentially serve each of these levels [10]. Generally, the existence of damage is detected by observing changes in the sensor signals in relation to any kind of baseline, e.g., predictions of analytical or numerical models or a baseline measurement for the pristine state of the structure [11]. For guided wave-based applications, typically, the baseline is subtracted from a current signal to isolate the scattered waves due to possible damage [12]. Furthermore, the position of damage can be estimated by analyzing the time-of-flight of the scattered guided waves for several actuator–sensor pairs [13]. Furthermore, Bayesian approaches can help to deal with uncertainties or to optimize sensor positions when localizing damage [14,15]. Eremin et al. [16] used an extended time-reversal approach to localize damages in anisotropic laminated composite structures and experimentally validated the approach using varying artificial surface obstacles.

Generally, the interaction of guided waves with damages includes complex phenomena, e.g., reflection, transmission, mode conversion, or generation of higher-harmonics, and is difficult to describe analytically. Analytical solutions are limited to simple damage shapes, e.g., circular holes with partial or complete through-thickness [17,18]. Hence, several semi-analytical solutions have been developed combining analytic approaches with finite element (FE) methods. The semi-analytical FE method calculates the wave propagation in waveguides with arbitrarily shaped cross-sections, which are invariant in propagation direction [19]. This method describes guided wave displacements by harmonic functions in the wave propagation direction coupled to FEs over the cross section. Spada et al. [20] used this semi-analytical FE method in a global-local approach to describe guided wave propagation computationally efficient in the global region, while a local FE model simulated the interaction with different defects of a skin-stringer. Similarly, the semi-analytical hybrid approach combines the spectral element method to effectively simulate the wave dynamics in a local area, while a semi-analytical boundary integral method describes the wave propagation in the global host structure [21]. Golub et al. [22] used this approach to investigate the scattering behavior of different damages in the bonding of a stringer on a thin plate and verified their results by experimental data. Another hybrid global–local approach is the combined analytical FE approach (CAFA) [23]. The CAFA calculates the wave propagation in the pristine global region by analytical solutions and simulates the complex interaction of guided waves with damage locally using a detailed FE model. This local FE model uses non-reflective boundaries (NRBs) to efficiently damp boundary reflections [24]. These NRBs enable an efficient harmonic simulation in the frequency domain and the scattered guided wave field due to the damage can be described by complex-valued wave damage interaction coefficients (WDICs). These WDICs describe the damage as a direction-dependent point source in the analytical framework. Bhuiyan et al. [25] investigated guided wave scattering at butterfly cracks in a multiple rivet row connection and analyzed sensible frequencies and sensing directions of these WDICs. Mei and Giurgiutiu [26] proposed a modal decomposition method to extract WDICs of high-order guided wave modes and investigated a through thickness hole and a circular sub-surface crack in an aluminum plate. A numerical study using a limited number of FE simulations shows the sensitivity of WDICs to such sub-surface cracks in aluminum for different normalized damage properties [27]. Migot et al. [13] analyzed different incident wave directions for through thickness cracks in aluminum plates and presented the WDIC scattering pattern for single frequencies. Shen and Cesnik [28] proposed the usage of WDICs to cover mode conversion and the higher harmonic generation phenomenon caused by nonlinear wave damage interaction, which is demonstrated by a breathing crack. Humer et al. [29] compared WDIC patterns of artificial damages for selected frequencies for numerical simulation results and experimental measurements using a scanning laser Doppler vibrometer (SLDV). The tremendous potential of WDICs as highly sensitive damage features that vary with the characteristics of the damage is demonstrated by Humer et al. [30]. This numerical study used a limited WDIC database of simulated damages and a simple damage metric to identify certain damage characteristics. Furthermore, it has been demonstrated that carefully selected deep neural networks can predict WDICs of damages that have not previously been simulated and, thus, extend such simulated WDIC databases substantially and highly efficiently [31].

However, the present study extends this previous research by presenting the amplitude and phase of complex-valued WDICs with a novel collective representation, which enables a holistic visual evaluation and comparison of results. This collective WDIC representation is used to comprehensively highlight their potential for model-based damage identification, but also points out issues with their high sensitivity to the smallest damage changes. In particular, the phase coefficients presented herein have usually not been analyzed. Furthermore, a detailed comparison between results of numerical and experimental investigations is presented for several damage scenarios by means of dimensionless damage parameters. Additionally, challenges for the experimental measurements of WDICs using an SLDV are revealed and possible solutions are outlined. Therefore, an improved baseline subtraction method is proposed to correct misalignment errors for the experimental measurements, which clearly enhances the resulting WDIC pattern. However, the presented comprehensive WDIC measurements by a single SLDV are not applicable to practical SHM applications. Sensor arrays are limited and thus, measurement noise and other uncertainties make damage identification challenging. In particular, numerical WDIC prediction of specific damages may be very challenging due to WDICs’ high sensitivity to structural changes. However, the presented paper is intended to support researchers and practitioners in ultrasonic based NDE or SHM to gain a comprehensive overview and pave the way for the finding of novel damage identification methods based on WDICs.

The structure of the paper is as follows. First, the underlying theory of the WDICs, the geometric scaling with the introduced dimensionless damage parameters, and the proposed improved baseline subtraction are described. Second, the numerical investigations and the used FE model are explained. Third, the experimental investigations using an SLDV to measure the scattered guided waves and necessary data processing steps are described. Fourth, a detailed comparison between WDIC predictions by these numerical simulations and experimental measurements is discussed. Finally, conclusions and future work from the present study are summarized.

## 2. Theory

For embedded ultrasonic NDE, typically, PWASs are attached to or even integrated into a structure to monitor its actual damage state. The PWASs can be used for excitation and/or sensing of guided waves [3]. These guided waves are ultrasonic elastic waves propagating in thin plates with complex behavior, e.g., multimodal and dispersive characteristics, which can be described by the Rayleigh-Lamb equation [32]. However, guided waves can propagate over large distances while interacting even with small discontinuities, e.g., damages, which makes them suitable for embedded ultrasonic NDE or SHM applications. The interaction of the incident guided waves with possible damage is quite complex even in an isotropic plate, and analytical solutions can only be found for simple cases, e.g., through thickness and partly through thickness holes [17] or thickness changes [33]. Therefore, the CAFA proposes to use detailed FE models to simulate the wave damage interaction [23]. These FE simulations reveal the characteristic scattering patterns arising for each damage scenario, which can be described analytically by WDICs. In the following section, these WDICs are explained in detail. Furthermore, the proposed dimensionless damage parameters are introduced by using the similitude theory for geometrically scaled scenarios.

### 2.1. Wave Damage Interaction Coefficients

If a probing guided wave packet travels through a structure and hits possible damage, the following interaction generates a characteristic scattering pattern due to constructive and deconstructive interference in the scattered wave field. This complicated wave damage interaction can be described analytically using WDICs, where the damage is approximated by a direction-dependent point source [23]. These complex-valued WDICs modify the amplitude and phase of the incident wave to result in the scattered waves, which can be mathematically formulated by

(1) $$\begin{array}{c} u_{B}^{\mathrm{SC}}\left(f,\gamma \right)e^{-i\varphi_{B}^{\mathrm{SC}}\left(f,\gamma \right)}=u_{A}^{\mathrm{IN}}\left(f\right)e^{-i\varphi_{A}^{\mathrm{IN}}\left(f\right)}C_{AB}\left(f,\gamma \right)e^{-i\varphi_{AB}\left(f,\gamma \right)}H_{1}^{\left(1\right)}\left(\xi_{B}\left(f\right)r_{\mathrm{sen}}\right). \end{array}$$

In Equation (1), the incident wave field has wave mode *A* and its amplitude and phase at the damage center position in the pristine state are described by $u_{A}^{\mathrm{IN}}\left(f\right)$ and $\varphi_{A}^{\mathrm{IN}}\left(f\right)$, respectively. Both depend on the frequency *f*. Analogously, the scattered waves with wave mode *B* are expressed by the term $u_{B}^{\mathrm{SC}}\left(f,\gamma \right)e^{-i\varphi_{B}^{\mathrm{SC}}\left(f,\gamma \right)}$, where *i* is the imaginary unit. The sensing points for the scattered waves around the damage are along a sensing circle with radius $r_{\mathrm{sen}}$ at certain sensing angles γ (typically defined with respect to the incident wave direction). The wave propagation from the point source at the damage center position to these sensing points with wavenumber $\xi_{B}\left(f\right)$ is described by the first kind Hankel function of first order $H_{1}^{\left(1\right)}\left(\xi_{B}\left(f\right)r_{\mathrm{sen}}\right)$. The complex interaction between guided waves and damage also excites evanescent waves in the near field around the damage, which typically covers only a couple of plate thicknesses away from the damage [34]. Hence, a sensing circle of sufficient size is crucial to only capture the propagating wave parts of the scattered wave field. However, besides the direct scattering, i.e., the incident and scattered waves having the same guided wave mode, the wave damage interaction might also cause mode conversion. This means that the incident wave mode *A* and scattered wave mode *B* are different, e.g., incident waves in fundamental symmetric mode $S_{0}$ can be converted to scattered waves in fundamental asymmetric mode $A_{0}$, and vice versa.

However, rearranging Equation (1) reveals the amplitude coefficients $C_{AB}\left(f,\gamma \right)$ as

(2) $$\begin{array}{c} C_{AB}\left(f,\gamma \right)=\left|\frac{u_{B}^{\mathrm{SC}}\left(f,\gamma \right)}{u_{A}^{\mathrm{IN}}\left(f\right)}\frac{1}{H_{1}^{\left(1\right)}\left(\xi_{B}\left(f\right)r_{\mathrm{sen}}\right)}\right| \end{array}$$

and phase coefficients $\varphi_{AB}\left(f,\gamma \right)$ as

(3) $$\begin{array}{c} \varphi_{AB}\left(f,\gamma \right)=\Delta \varphi_{AB}\left(f,\gamma \right)-\left[∠\left(\frac{1}{H_{1}^{\left(1\right)}\left(\xi_{B}\left(f\right)r_{\mathrm{sen}}\right)}\right)-∠\left(\frac{1}{H_{1}^{\left(1\right)}\left(0^{+}\right)}\right)\right]. \end{array}$$

The amplitude coefficients in Equation (2) are calculated using the absolute value |·|. In Equation (3), the operator ∠(·) determines the phase of its argument. The function $H_{1}^{\left(1\right)}\left(0^{+}\right)$ is evaluated at the radius $r=0^{+}$, which is the point source position respectively the origin of the cylindrical coordinate system approached from the positive side.The phase difference $\Delta \varphi_{AB}\left(f,\gamma \right)$ in Equation (3) is calculated between the phase of the incident waves $\varphi_{A}^{\mathrm{IN}}\left(f\right)$ and the phase of the scattered waves $\varphi_{B}^{\mathrm{SC}}\left(f,\gamma \right)$ by

(4) $$\begin{array}{c} \Delta \varphi_{AB}\left(f,\gamma \right)=\varphi_{B}^{\mathrm{SC}}\left(f,\gamma \right)-\varphi_{A}^{\mathrm{IN}}\left(f\right). \end{array}$$

In this paper, the focus is on the wave damage interaction of the fundamental asymmetric $A_{0}$ wave mode due to two reasons. First, this mode can be measured more easily by a single LSDV in experimental investigations. Second, the shorter wavelength compared to the fundamental symmetric $S_{0}$ wave mode makes it more sensitive to small damages. Hence, the amplitude coefficients in Equation (2) and phase coefficients in Equation (3) are evaluated only for an incident $A_{0}$ to a scattered $A_{0}$ wave mode, i.e., $A=A_{0}$ and $B=A_{0}$. For the sake of simplicity, from now on, the resulting amplitude coefficients $C_{A_{0}A_{0}}$ and phase coefficients $\varphi_{A_{0}A_{0}}$ are abbreviated as *C* and φ, respectively.

The scattered waves caused by the damage are isolated using the baseline subtraction method [12], where the baseline measurement of the incident wave field in the pristine case is subtracted from the total wavefield in the damaged case. Therefore, the displacement of the scattered wave field $u^{\mathrm{SC}}$ is calculated by

(5) $$\begin{array}{c} u^{\mathrm{SC}}=u^{\mathrm{TOT}}-u^{\mathrm{IN}} \end{array}$$

using the incident wave displacements $u^{\mathrm{IN}}$ and the total wave displacements $u^{\mathrm{TOT}}$. Furthermore, a decomposition given by

(6) $$\begin{array}{c} \begin{array}{cc} u_{S_{0}} & =\frac{u_{\mathrm{Top}}-u_{\mathrm{Bot}}}{2}\\ u_{A_{0}} & =\frac{u_{\mathrm{Top}}+u_{\mathrm{Bot}}}{2} \end{array} \end{array}$$

reveals the fundamental symmetric and asymmetric parts of these wave fields for an isotropic plate. In Equation (6), the out-of-plane displacements $u_{\mathrm{Top}}$ and $u_{\mathrm{Bot}}$ at the top and bottom surface of the investigated structure are used, respectively. This is to say that the wave decomposition in Equation (6) would be exactly the opposite for the in-plane-displacements. However, the presented formulas in Equations (1)–(6) can be used analogously for the out-of-plane velocity *v*, e.g., measured by a single SLDV, by substituting the out-of-plane displacement *u*. Generally, the wave damage interaction can also cause higher guided wave modes in the scattered wave field. To cover such mode conversions for the WDICs, a more comprehensive decomposition is required, which takes the different non-linear mode shapes in thickness direction into account [26]. However, for the investigated plate thicknesses and the selected frequencies, only the fundamental guided wave modes exist. Therefore, herein, the simple decomposition in Equation (6) is sufficient.

### 2.2. Geometrical Scaling

In general, the guided wave scattering of damage depends on both the characteristics of the guided waves and the damage. First, the guided wave characteristics for a isotropic plate are basically determined by the Rayleigh–Lamb equation [32]. The solution of this transcendental equation for selected material parameters gives the wavenumber and from it, further guided wave characteristics, e.g., wavelength λ, can be easily derived. Second, the characteristics of the damage mainly depend on the damage type and its descriptive parameters. However, the dimensions of each particular damage are described by geometric parameters. The present study investigates structural discontinuities similar to damages introduced by miniature steel sheets of square shape bonded to the top side of an aluminum plate. The geometric parameters of such artificial damage are the damage size *D*, the damage thickness *t*, and the damage orientation α. Together with the wavelength λ, which depends on the frequency *f*, these geometric parameters define the wave damage interaction uniquely and the WDICs can be calculated in a certain frequency range for given material parameters. However, the question is whether the WDICs are similar for a thicker plate, but also for larger damage and wavelength, respectively. Generally, by using similitude theory and considering a geometrically scaled scenario, the physical dimensions can be replaced by dimensionless parameters. Therefore, the WDIC results are not only valid for a single damage scenario, but simultaneously for all scaled scenarios with identical dimensionless parameters. This fact enables a more universal use of the WDICs, which tremendously reduces the time for numerically simulating and/or experimentally measuring. However, for the selected artificial damages, the dimensionless parameters

(7) $$\begin{array}{c} \begin{array}{cc} \tau & =\frac{t}{h}=\frac{t}{2d}\\ \delta & =\frac{D}{t}\\ \Lambda & =\frac{D}{\lambda } \end{array} \end{array}$$

are proposed. In Equation (7), the dimensionless damage to plate thickness ratio τ, the dimensionless damage size to damage thickness ratio δ, and the dimensionless damage size to wavelength ratio Λ are defined. These three dimensionless parameters relate the four physical dimensions and thus describe the geometric relations uniquely for each scaled scenario.

In the following, the use of these dimensionless parameters to compare the results of two different scenarios is explained in more detail. For the first scenario, a given combination of a plate and a damage defines the dimensionless parameters by using Equation (7), and thus the WDICs can be calculated for certain frequencies *f*, assuming the second scenario to be geometrically similar and scaled by a certain factor. This means the plate thickness changes, and so does the wavelength compared to the first scenario for the same frequency. Thus, the comparison of the WDIC results have to be compared for the characteristic product of frequency and half plate thickness. By doing so, the dimensionless damage size to wavelength ratio Λ is equal for both scenarios. Furthermore, the auxiliary parameter Λ is an approximate indicator for the sensibility of the probing guided wave for a certain damage size. For very low values of Λ, the scattering of the damage might be very low as well, and the damage may possibly remain undetected. In such cases, it could help to increase the frequency, i.e., reducing the wavelength λ, to raise the scattered wave amplitudes.

**Figure 1:**
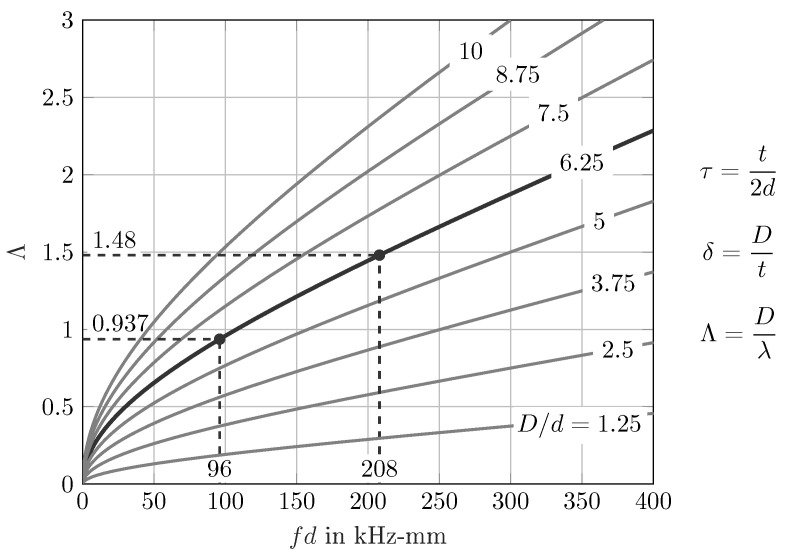
Dimensionless damage size to wavelength ratio Λ for different ratios D/d.

However, in Figure 1, the dimensionless parameter Λ is plotted over the product of frequency *f* and half plate thickness *d* for different damage scenarios.

On the one hand, this figure can help to estimate the value of Λ for a certain range of fd or vice versa. On the other hand, the influence of the ratio D/d (damage size *D* to half plate thickness *d*) can be clearly seen. Additionally, the region for the later discussed WDIC comparison of the scaled numerical and experimental scenarios is highlighted.

### 2.3. Improved Baseline Subtraction and Time History Tapering

For guided wave applications, the scattered wavefield caused by the wave damage interaction is typically isolated by using the baseline subtraction method; see Equation (5). In this paper, the scattered guided waves around the damage and the resulting WDICs are evaluated for numerical simulations and experimental measurements. This is to say that a single SLDV measures the out-of-plane velocities of the guided waves in the experiments, whereas the out-of-plane displacements are extracted from the numerical simulation results. However, the presented equations for baseline subtraction, wave decomposition, and WDIC calculation in Equations (2)–(6) can be used analogously by substituting the displacement *u* by the velocity *v*.

For the experimental measurements, the SLDV needs to be aligned to the plate for every measurement, e.g., measuring on the top side of the plate in the pristine and damaged scenario. This realignment causes a small positioning error of the plate and consequently a time shift in the measured guided wave signal. Hence, for the damaged case, the baseline subtraction does not remove the incident waves perfectly. To address this systematic measurement error, the baseline subtraction needs to be adjusted for each measurement and an improved baseline subtraction method is proposed.

Generally, the SLDV alignment to the plate causes a positioning error in the *x*- and *y*-directions. Due to the dominant wave propagation in the *x*-direction, i.e., the nearly straight-crested wave front arriving at the damage, a positioning error in the *y*-direction moves the scanning point parallel to the wave crest. This causes a negligible difference for the wave amplitude, while a minor misalignment in the *x*-direction causes significant amplitude changes. Hence, the positioning error in the *y*-direction affects the baseline subtraction marginally, and only the error in *x*-direction is corrected. For this correction, a part of the incident wave in the total wavefield of the damaged scenario is selected and time-shifted to align with the baseline measurement. Therefore, several points within one wavelength of the incident guided wave need to be measured in the *x*-direction between the PWASs and the damage position. Then the residuum

(8) $$\begin{array}{c} r\left(\Delta t\right)=\sqrt{\sum_{x,t}{\left(v\left(x,t+\Delta t\right)-v_{B}\left(x,t\right)\right)}^{2}} \end{array}$$

is calculated for the current and the baseline wavefields *v* and $v_{B}$, respectively, of these points in a selected time range. This time range has to cover a part of the incident wave in the total wavefield of the damaged scenario that is not influenced by any reflections from the damage. This is shown in detail for an example in Section 4.2. However, the time shifts Δt are selected to cover the expected positioning error of the plate in both directions, i.e., for negative and positive values. In Equation (8), *v* is the current wavefield to be time shifted, e.g., a damage scenario measured on the top side, and $v_{B}$ is the baseline, e.g., a pristine scenario measured on the top side. The residuum *r* results in a convex function over the time shifts Δt. The minimum of this function indicates the optimal time shift to correct the positioning error of the plate in the *x*-direction for the current measurement. By applying this time shift to all measurement points of the current wavefield and then subtracting the baseline measurement, the residual incident waves in the scattered wavefield are significantly reduced. Therefore, this improved baseline subtraction method based on such time shifts is used herein to correct the positioning error of the plate. In contrast to other methods, e.g., global space–time alignment [35], the proposed improvement of the baseline subtraction needs only a few scan points instead of a time-demanding full wavefield scan. Therefore, it might be useful in ultrasonic-based NDE applications, e.g., to calibrate laboratory measured baseline data to measurements in real systems or for automated series production using contactless measurement systems such as an SLDV.

However, for the experimental measurements, the improved baseline subtraction method reveals the isolated scattered wavefield around the damage, i.e., the out-of-plane velocities $v_{\mathrm{Top}}^{\mathrm{SC}}$ and $v_{\mathrm{Bot}}^{\mathrm{SC}}$ on the top and bottom of the plate, respectively. By using Equation (6), these waves can be decomposed into asymmetric $v_{A_{0}}^{\mathrm{SC}}$ and symmetric $v_{S_{0}}^{\mathrm{SC}}$ parts. Analogously, the incident wave fields $v_{A_{0}}^{\mathrm{IN}}$ and $v_{S_{0}}^{\mathrm{IN}}$ at the equivalent damage position in the pristine case can be calculated. However, before using these decomposed scattered and incident waves for WDIC calculations, an additional filter is applied for time history tapering [36]. Hence, discontinuities at the beginning and the end of the measured time signals are suppressed and leakage problems for the following Fourier transform are avoided. In the present study, a raised cosine filter

(9) $$\begin{array}{c} H\left(t\right)=\left\{\begin{array}{cc} 1 & \mathrm{for}\;\left|t\right|\leq \frac{T_{F}}{2}\\ \frac{1}{2}\left(1+\cos\left(\frac{\pi }{T_{R}}\left[\left|t\right|-\frac{T_{F}}{2}\right]\right)\right) & \mathrm{for}\;\frac{T_{F}}{2}<\left|t\right|\leq \frac{T_{F}}{2}+T_{R}\\ 0 & \mathrm{otherwise}\; \end{array}\right. \end{array}$$

is defined in the time domain. It helps to suppress measurement noise and to further improve the signal quality. In Equation (9), the filter width $T_{F}$ and the raise time $T_{R}$ can be used to adapt the filter, e.g., to different excitation signals. The application of this filter is presented in Section 4.2 in detail.

## 3. Numerical Simulations

Numerical simulation models are valuable to study guided wave scattering at damages due to high computing power that is readily available. Such simulations offer high flexibility when investigating the influence of different parameters or complex damage shapes on the scattering behavior. Furthermore, approximate solutions can be numerically calculated even if analytical solutions are not available. In the present study, a numerical model based on the FE method in the commercial software ABAQUS 2018 [37] is used.

### 3.1. Finite Element Model

**Figure 2:**
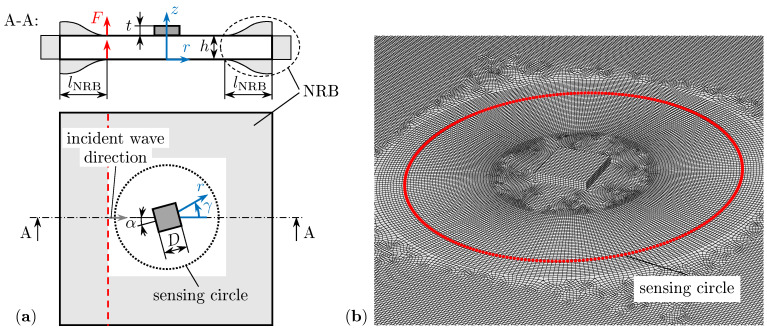
Local FE model: (**a**) sketch; (**b**) mesh in the center region with sensing nodes ($r_{\mathrm{Sen}}=30\;\mathrm{mm}$).

The FE model efficiently simulates the guided wave scattering in detail and enables the extraction of WDICs as proposed by the CAFA [23]; see Figure 2.

The focus of this simulation model is on the damaged area and its immediate surrounding of the investigated structure. The damages are introduced by surface bonded miniature steel sheets of square shape with a size of D=10mm and selected thickness ratios $\tau =\frac{t}{h}$ and orientation α, respectively. For an assumed ideal bonding between the damage and the host structure the nodes in the contact area are tied together. The host structure is an aluminum plate with thickness h=1.6mm. The material properties for the damage and the plate are listed in Table 1.

The plate boundaries are completely covered by NRBs to suppress unwanted reflections and, thus, a plate of infinite size is simulated. Furthermore, these NRBs enable simulations using harmonic excitation directly in the frequency domain, the so-called steady-state dynamics (SSD) analysis. The combination of this SSD analysis and the compact model size ($150\times 150\times 1.6\;{\mathrm{mm}}^{3}$) due to the utilized NRBs allows efficient WDIC simulations. For sufficient damping, the NRBs should cover at least two times the length of the longest wavelength [24]. In the present model, the NRBs are applied using a half Von-Hann window function shape over a length $l_{\mathrm{NRB}}=30\;\mathrm{mm}$ with a damping value ζ=0.3 as recommended by Shen and Giurgiutiu [24]; see Figure 2a.

The guided waves are excited by two linear line loads (wave field curvature can be neglected due to the assumed large distance to the actuator PWAS) on the top and bottom of the plate on the left side of the damage. The wave damage interaction is simulated for a frequency range from 80kHz to 320kHz in steps of 2kHz.

Generally, for wave propagation simulations, a relatively fine mesh with at least ten elements per wavelength is recommended [38]. Therefore, the plate is meshed with a maximum in-plane size of 0.5mm and the thickness direction is discretized with four elements; see Figure 2b. The selected eight-node linear solid elements C3D8 use full integration. In total, the FE model contains approximately 810,000 elements and needs approximately 7h on a standard desktop computer. The guided wave displacements are evaluated for the out-of plane direction at sensing nodes every 1.25 ° along the sensing circle for the pristine and selected damage models; see Figure 2b. The radius of the sensing circle $r_{\mathrm{sen}}=30\;\mathrm{mm}$ is selected to capture only the propagating wave parts in the far-field of the scattered waves. Additionally, the out-of plane displacements are extracted at the equivalent damage center position in the pristine model for the top and bottom nodes, respectively. Hence, using the baseline subtraction and wave decomposition in Equations (5) and (6), respectively, reveals the asymmetric scattered wave displacements $u_{A_{0}}^{\mathrm{SC}}$ and the asymmetric incident wave displacement $u_{A_{0}}^{\mathrm{IN}}$, respectively. These displacements are complex-valued (due to the SSD analyses) and the phases $\varphi_{A_{0}}^{\mathrm{SC}}$ and $\varphi_{A_{0}}^{\mathrm{IN}}$ are simply defined by their arguments. Therefore, the amplitude and phase coefficients *C* and φ can be calculated using Equations (2)–(4) for every sensing angle γ in the simulated frequency range. The WDICs depend on the properties of the damage and the host structure. The latter remains constant in this study and only the influence of the dimensionless thickness ratio τ and orientation α on the WDICs are investigated herein. However, the numerical study could easily be extended.

### 3.2. Numerical WDICs

In the present study, the WDICs, i.e., the amplitude coefficients *C* and phase coefficients φ, are investigated for a guided wave scattering from an incident asymmetric to a scattered asymmetric guided wave mode. The WDICs are presented by means of dimensionless parameters. Therefore, the WDIC results of the simulated combination of damage and structure can easily be converted to a geometrically similar scenario of a different scale. In the present study, three dimensionless parameters, namely, the dimensionless thickness ratio τ, the dimensionless damage size to thickness ratio δ and the dimensionless damages size to wavelength ratio Λ, allow the use of the WDIC results in an universal way for all scaled scenarios. To study the influence of different damage characteristics on the WDICs, twelve simulations for τ∈{0.5,0.75,1} and $\alpha \in \{0^{{}^{\circ}},15^{{}^{\circ}},30^{{}^{\circ}},45^{{}^{\circ}}\}$ are investigated.

However, the numerical FE models are used to simulate the wave scattering in a frequency range from 80kHz to 320kHz for each damage scenario. This frequency range is converted to a range of 64kHz-mm to 256kHz-mm for the characteristic product fd by taking into account the half plate thickness d=0.8mm. Thus, the dependency of the wavelength λ on the half plate thickness *d* is considered and equal wavelength ratios Λ are guaranteed in this fd-range when comparing results of scaled scenarios.

**Figure 3:**
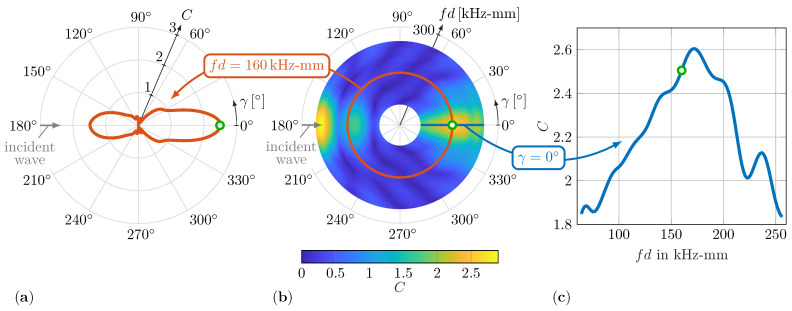
Numerical simulation results of the amplitude coefficients *C* for $\alpha =0^{{}^{\circ}}$ and τ=0.5: (**a**) scattering pattern for fd=160kHz-mm; (**b**) amplitude coefficients *C* for all combinations of fd and γ; (**c**) amplitude coefficients *C* for constant sensing angle $\gamma =0^{{}^{\circ}}$.

Typically, the WDIC results are presented in two different ways. First, they are presented for a constant sensing angle γ to highlight sensible values for fd, as depicted in Figure 3c.

Second, the scattering pattern for a constant value of fd reveals sensible sensing directions γ; see Figure 3a. To display all information of the amplitude coefficients *C* in a single plot, a novel collective representation, shown in Figure 3b, is proposed. From this graph, the influence of the damage characteristics on the amplitude coefficients *C* can be observed simultaneously for the sensing angle γ and the product fd, thus enabling a better comparison of the WDIC patterns. Therefore, the sensing angle γ is plotted in the angular direction and the product fd in the radial direction, while the value of the corresponding amplitude coefficient *C* is color-coded. The wave damage interaction and its resulting scattering patterns relate to the incident wave direction, which is indicated by an arrow; see Figure 3a,b.

The numerical results of the phase coefficients φ can be displayed analogously. They are presented together with the amplitude coefficients *C* in Section 5.1 with a detailed discussion on the influence of selected damage properties τ and α. Furthermore, a direct comparison to experimental results is shown.

## 4. Physical Experiments

The scattering of ultrasonic guided waves due to damages is demonstrated by a laboratory experiment using a single SLDV. From the SLDV measurements of a pristine and damaged scenario, the scattered wavefield around the damage and, with it, the WDICs, are extracted and compared to numerical results.

### 4.1. Experimental Setup

**Figure 4:**
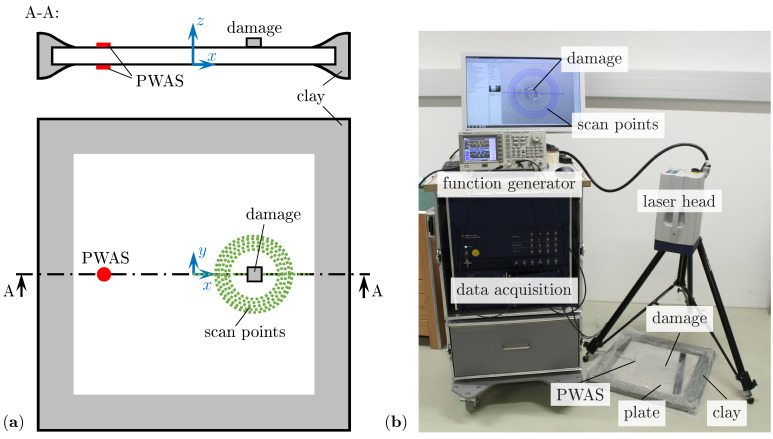
Physical experiments: (**a**) sketch; (**b**) experimental setup using an SLDV.

As host structure, a thin aluminum alloy EN AW-5754 plate, as typically used for lightweight structures, with dimensions $500\times 500\times 2\;{\mathrm{mm}}^{3}$, is selected. For this structure, only the fundamental symmetric $S_{0}$ and asymmetric $A_{0}$ wave modes exist in the investigated frequency range below 300kHz. The utilized single SLDV (Polytech PSV-500-HV) measures the out-of-plane velocity of the guided waves around the damage position. Therefore, this study focuses on the asymmetric $A_{0}$ wave mode due to its dominant out-of-plane motion in comparison to the symmetric $S_{0}$ mode. The guided waves are excited by two circular PWASs made from PIC 255 material [39] with dimensions $ø10\times 0.2\;{\mathrm{mm}}^{2}$ and wrapped around electrodes. These PWASs are glued opposite to each other on the top and bottom side of the plate at the position x=−100mm using structural epoxy; see Figure 4a.

These actuator PWASs are asymmetrically excited, i.e., the excitation signal for the bottom PWAS is inverted to predominantly excite the asymmetric $A_{0}$ wave mode. As an excitation signal, a typical burst with a five period Von-Hann windowed sine function with a peak-to-peak amplitude $V_{\mathrm{PP}}=18\;V$ is provided directly by an arbitrary function generator. To cover a broader frequency range, two burst signals with central frequencies of $f_{c}=130\;\mathrm{kHz}$ and $f_{c}=200\;\mathrm{kHz}$ are used successively for every damage scenario.

However, the excited guided waves travel through the plate to the damage position $x_{D}=50\;\mathrm{mm}$, where the damage scatters the incident waves. As artificial damages, miniature steel sheets of square shape with varying thickness ratio τ and orientation α are bonded to the top surface of the plate using regular superglue. This type of damage can be easily removed and reattached, e.g., with different orientations α, as is common practice in laboratory experiments [16,40]. The boundaries of the plate are covered by clay specifically shaped to suppress unwanted reflections; see Figure 4. The areas on the top and bottom of the plate near the damage position, which are measured by the SLDV, are additionally treated with a reflective spray. This ensures a uniform reflectivity for all scan points and minimizes measurement noise due to the laser.

Generally, the complex interaction of the interrogating guided waves with the damage might also include mode conversion, i.e., an incident asymmetric $A_{0}$ wave is scattered into a symmetric $S_{0}$ wave or vice versa [26]. Hence, a differentiation between symmetric and asymmetric modes of the incident and scattered waves is mandatory. This is realized by measurements from the top and the bottom sides of the plate and a subsequent wave decomposition. Therefore, the plate needs to be repositioned in relation to the SLDV, which is then realigned with an accuracy of approximately 0.1mm. This systematic positioning error is addressed by using the proposed improved baseline subtraction method and its influence is discussed in detail below. However, the SLDV uses a sampling frequency $f_{s}=6.25\;\mathrm{MHz}$ to resolve the guided wave signals over a time period T=160μs. For each scan point, 1000 measurements are averaged to minimize noise. Furthermore, a bandpass filter with cut-off frequencies of 30kHz and 400kHz suppresses low frequency vibrations from the surroundings and high frequent noise from the measurement equipment. The SLDV measures the guided waves from the center of the plate, i.e., the origin of the plate coordinate system, along the *x*-axis up to x=100mm with a resolution of 1mm; see Figure 4a. This line scan is used to check the damping effect of the clay on the boundary reflections and the improved baseline subtraction. Additionally, five sensing circles with radii from 30mm to 40mm in steps of 2.5mm with sensing points each $2.5^{{}^{\circ}}$ centered at the damage position are investigated; see Figure 4a. Hence, a possible dependency of the WDIC patterns on the sensing circle size is studied.

### 4.2. Experimental WDICs

**Figure 5:**
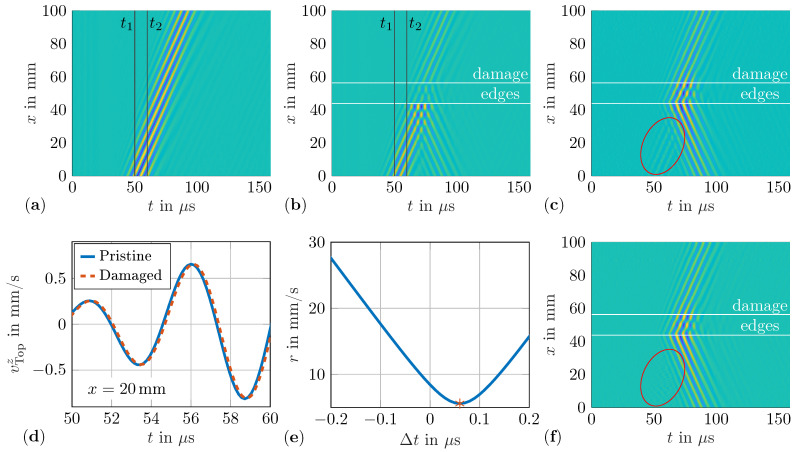
Measured out-of-plane velocity $v_{\mathrm{Top}}$ along the *x*-axis on the top side of the plate for a burst with $f_{c}=200\;\mathrm{kHz}$: (**a**) pristine case (baseline); (**b**) damaged case (τ=1 and $\alpha =0^{{}^{\circ}}$); (**c**) scattered waves; (**d**) selected signals (between $t_{1}=50\;\mu m$ and $t_{2}=60\;\mu m$) for baseline correction at x=20mm; (**e**) residuum *r* with indication of optimal time shift; (**f**) scattered waves using improved baseline subtraction.

The raw measurement data from the SLDV needs further data processing before the final WDIC calculation, i.e., baseline subtraction, wave decomposition, and additional filtering, can be performed. This is to say that for the experiment, the out-of-plane velocities measured by the SLDV are used in contrast to the out-of-plane displacements extracted from the numerical simulations. However, the formulas in Equations (2)–(6) can be used analogously by substitution. To isolate the scattered wave field from the total wave field in the damaged case, the baseline measurement needs to be subtracted as in Equation (5). This baseline subtraction method is visualized in Figure 5. Figure 5a shows the baseline measurement, Figure 5b the total wavefield for the damaged scenario with a thickness ratio τ=1 and orientation $\alpha =0^{{}^{\circ}}$, and Figure 5c the isolated scattered wave field along the *x*-axis. The wavefield along the *x*-axis for the pristine scenario can also reveal possible reflections from the plate boundaries. Figure 5 shows only the forward propagating incident wave packet; thus, sufficient attenuation by the clay is indicated.

In Figure 5b–d, the position of the damage edges are indicated by horizontal lines. The area between these lines shows the measurement results for the scanning points on top of the damage instead of on the top of the plate. It can be seen that the incident wave field is partly reflected and transmitted by the first edge of the damage. The latter propagates in the damaged region and is split up in reflected and transmitted waves by the second edge as well. Therefore, some of the wave energy is trapped within the damaged area. Furthermore, the scattered waves at the sensing points along the sensing circle are a superposition of these two scattering phenomena, and thus the WDIC describes them as a single scattering phenomenon.

However, due to the new alignment of the SLDV for every measurement and the resulting positioning error, the baseline subtraction does not remove the incident waves perfectly. Hence, a residual wave field remains in the scattered wave field, which clearly belongs to the baseline measurement; see the marked area in Figure 5c. To address this effect of the systematic measurement error, the baseline subtraction needs to be adjusted for each measurement. As already mentioned, only the positioning error in the *x*-direction is corrected due to the dominant wave propagation in this direction, i.e., the nearly straight-crested wave fronts. For this correction, parts of the incident waves in the total wavefield of the damaged scenario are selected and time-shifted to align with the baseline measurement. For this alignment, the measurements between t=50μs and t=60μs are selected as highlighted in Figure 5a,b. This time period is selected as there is no reflection from the damage in the signal, i.e., the undisturbed incident wave is also measured for a plate with damage. For example, Figure 5d shows the measurements of the pristine and damaged scenario on the top side of the plate at x=20mm. The resulting time shift due to the positioning error of the plate can clearly be seen in this time range. These measurements are resampled with a refined time resolution of 10ns, allowing more accurate position corrections in the expected range of 0.1mm. Then, the residuum *r* is calculated by Equation (8) for all measured points along the *x*-axis in the selected time range. The time shifts Δt are selected to cover the expected positioning error of the plate in both directions, i.e., for negative and positive values. In Equation (8), *v* is the current wavefield to be time shifted, e.g., a damage scenario measured on the top side, and $v_{B}$ is the baseline, e.g., a pristine scenario measured on the top side, as illustrated in Figure 5a,b, respectively. The residuum *r* results in a convex function over the time shifts Δt; see Figure 5e. The minimum of this function indicates the optimal time shift to minimize the positioning error of the plate in the *x*-direction for the current measurement. For the example, illustrated in Figure 5, the optimal time shift is 60ns, which corresponds to a position correction of 0.166mm in the expected range using an approximated group velocity of 2780m/s. Figure 5f shows the results of the scattered waves using the time shifted signal for the measurement on the top side of the plate in the damaged case, i.e., the improved baseline subtraction. Comparing the highlighted areas in Figure 5c,f clearly illustrates the significantly lower residual wave field when using this improved baseline subtraction method.

However, four measurement results, i.e., the top and bottom of the pristine and damaged scenarios, are necessary to isolate the scattered waves for each damage type and excitation signal. Hence, the pristine scenario is measured for each of the two excitation signals on the top and bottom of the plate. This measurement procedure is also applied to the twelve damaged scenarios, i.e., all combinations of τ∈{0.5,0.75,1} and $\alpha \in \{0^{{}^{\circ}},15^{{}^{\circ}},30^{{}^{\circ}},45^{{}^{\circ}}\}$. Thus, in total, 52 experimental measurements are performed. However, for the improved baseline subtraction, the pristine measurement on the top side of the plate is taken as reference; i.e., the baseline wavefield $v_{B}$ in Equation (8), and the remaining are optimally time shifted according to it. For the alignment of the measurements on the bottom side of the plate, the results need to be inverted before calculating the residuum due to the asymmetric wave motion of the investigated $A_{0}$ mode.

**Figure 6:**
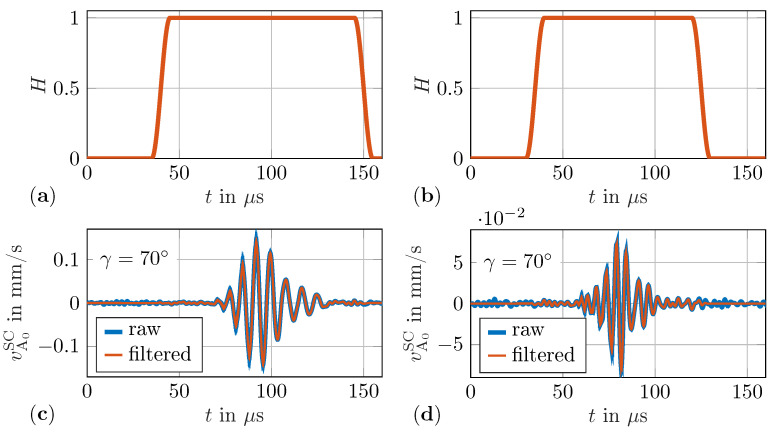
Raised cosine filter for experiments using (**a**) 130kHz burst excitation and (**b**) 200kHz burst excitation. Raw and filtered signals for $\gamma =70^{{}^{\circ}}$ and $r_{\mathrm{Sen}}=30\;\mathrm{mm}$ (**c**) 130kHz burst excitation and (**d**) 200kHz burst excitation.

The improved baseline subtraction method reveals the out-of-plane velocities $v_{\mathrm{Top}}^{\mathrm{SC}}$ and $v_{\mathrm{Bot}}^{\mathrm{SC}}$ of the scattered waves on the top and bottom of the plate, respectively. These waves can be decomposed into asymmetric $v_{A_{0}}^{\mathrm{SC}}$ and symmetric $v_{S_{0}}^{\mathrm{SC}}$ parts by using Equation (6). Analogously, the incident wave fields $v_{A_{0}}^{\mathrm{IN}}$ and $v_{S_{0}}^{\mathrm{IN}}$ at the equivalent damage position in the pristine case can be calculated. However, before using these decomposed scattered and incident waves for WDIC calculations, an additional filter is applied. In contrast, for the selected experimental setup, the measured guided wave signals are relatively low due to the low excitation signal. These signal amplitudes could be easily increased by using an amplifier to excite the PWASs with more energy. However, the raised cosine filter in Equation (9) is applied to the scattered guided wave signals for two excitation signals using central frequencies $f_{c}=130\;\mathrm{kHz}$ and $f_{c}=200\;\mathrm{kHz}$, respectively. Hence, two different filters are defined accordingly using a raise time $T_{R}=10\;\mu s$, where for the former the filter is centered at 95μs with a width $T_{F}=100\;\mu s$; see Figure 6a, while for the latter, the center is at 80μs with a width $T_{F}=80\;\mu s$; see Figure 6b. In Figure 6c,d, the raw and filtered versions of the scattered asymmetric velocity $v_{A_{0}}^{\mathrm{SC}}$ measured at a sensing angle $\gamma =70^{{}^{\circ}}$ and a sensing radius $r_{\mathrm{sen}}=30\;\mathrm{mm}$ are plotted for the excitation signal with central frequencies of 130kHz and 200kHz, respectively. By applying these raised cosine filters, the amplitudes at the beginning and the end of the filtered signals are zero. This time history tapering generates continuous periodic signals as assumed by the following Fourier transform and suppresses leakage problems.

**Figure 7:**
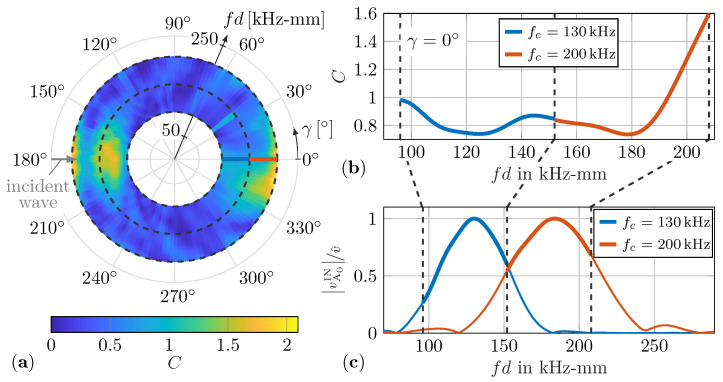
Experimental results for τ=0.5 and $\alpha =0^{{}^{\circ}}$ at $r_{\mathrm{Sen}}=30\;\mathrm{mm}$: (**a**) amplitude coefficients *C* for all combinations fd and γ; (**b**) amplitude coefficients *C* for $\gamma =0^{{}^{\circ}}$; (**c**) spectra of the incident asymmetric waves $v_{A_{0}}^{\mathrm{IN}}$ normalized by $\hat{v}=\max\left(\left|v_{A_{0}}^{\mathrm{IN}}\right|\right)$.

Finally, the filtered time signals for the asymmetric incident wave $v_{A_{0}}^{\mathrm{IN}}$ and asymmetric scattered waves $v_{A_{0}}^{\mathrm{SC}}$ are Fourier transformed to the frequency domain and inserted into Equations (2)–(4) for WDIC calculations. In Figure 7c, the frequency spectra for the normalized out-of-plane incident wave velocity $v_{A_{0}}^{\mathrm{IN}}$ for the two excitation bursts using central frequencies $f_{c}=130\;\mathrm{kHz}$ and $f_{c}=200\;\mathrm{kHz}$, respectively, are plotted over fd, the product of frequency *f* and half plate thickness d=1mm for the experiments.

The maxima of these spectra are below the fd values according to these central frequencies, i.e., fd=130kHz-mm and fd=200kHz-mm, due to the strongly dispersive behavior of the asymmetric $A_{0}$ guided wave mode; see Figure 7c. However, the windowed excitation signal in the time domain causes its frequency spectra to have zero points and the excited frequencies have different intensities; see Figure 7c. In Equation (2), the incident wave $v_{A_{0}}^{\mathrm{IN}}$ is inserted in the denominator for normalization and the resulting amplitude coefficients *C* approach infinity at these zero points. Therefore, the WDICs are evaluated only for values near the maxima of the frequency spectra to guarantee sufficient excitation. The two excitation signals are selected to overlap in the frequency domain and thus increase the usable range of fd values for the WDICs. In this study, the experimental WDIC results of the successive measurements using these two excitation signals are presented side by side; see Figure 7a,b. These results are investigated in the selected ranges from 96kHz-mm to 152kHz-mm and from 152kHz-mm to 208kHz-mm, respectively. In terms of the dimensionless damage size to wavelength ratio, a range from Λ≅0.94 to Λ≅1.48 is covered; see Figure 1. In Figure 7b, the difference of the results for the amplitude coefficients *C* for a sensing angle $\gamma =0^{{}^{\circ}}$ at a sensing radius $r_{\mathrm{Sen}}=30\;\mathrm{mm}$ is negligible for the two excitation signals at the intersection point at fd=152kHz. On the one hand, this indicates a sufficient overlapping of the excitation signals and, on the other hand, sufficient excitation of both excitation signals at this point. However, the results for the phase coefficients φ are illustrated analogously. They are presented together with the amplitude coefficients *C* and in comparison to the numerical results in the following discussion.

## 5. Data Analysis and Discussion

In this section, a detailed comparison between the numerical and experimental WDIC results is presented. The amplitude and phase coefficients *C* and φ, respectively, are directly compared using the proposed dimensionless parameters. The latter enable the comparison of the different but geometrically similar damage scenarios of the numerical and experimental investigations. However, the influence of the selected damage parameters, i.e., thickness ratio τ and orientation α, on the highly sensitive WDIC patterns are analyzed using the proposed novel collective representation. Furthermore, the dependency of the WDIC pattern, first on the advanced baseline subtraction, second on the size of the sensing circle, and third on the adhesive layer between the damage and the plate, is discussed.

### 5.1. Comparison of Numerical and Experimental WDICs

**Figure 8:**
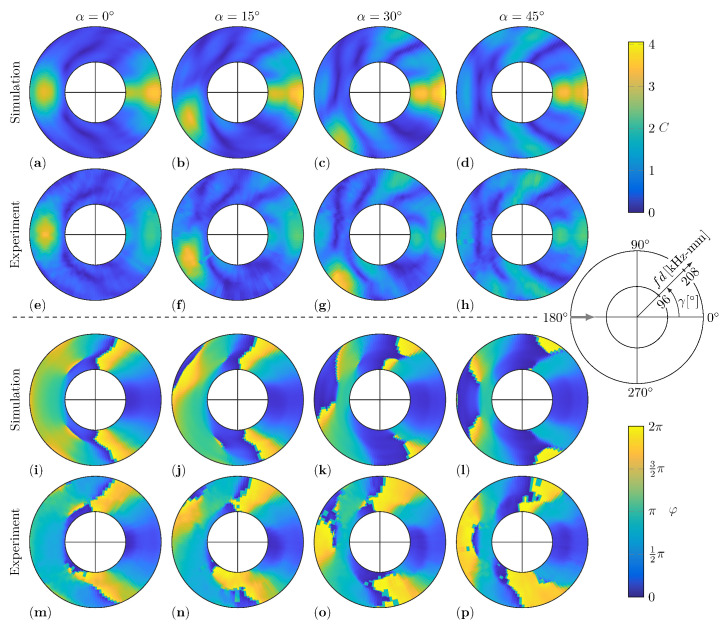
Comparison of WDIC results for τ=1 and different orientations α: (**a**–**d**) numerical amplitude coefficients; (**e**–**h**) experimental amplitude coefficients; (**i**–**l**) numerical phase coefficients; (**m**–**p**) experimental phase coefficients; (arrow in the coordinate system indicates incident wave direction).

In the present study, the WDIC predictions by numerical simulations and experimental measurements are compared for twelve damage scenarios. The investigated aluminum plates have different thicknesses for the numerical FE model and the experimental setup, i.e., t=1.6mm and t=2mm, respectively. By using the dimensionless parameters, namely the dimensionless damage to plate thickness ratio τ, the dimensionless damage size to damage thickness ratio δ and the dimensionless damage size to wavelength ratio Λ (see Equations (7)), these results can be directly compared over the characteristic product fd. Hence, the twelve damage scenarios are described by the combinations of τ∈{0.5,0.75,1} and $\alpha \in \{0^{{}^{\circ}},15^{{}^{\circ}},30^{{}^{\circ}},45^{{}^{\circ}}\}$. This is to say that all presented experimental WDIC results in this section are calculated using the improved baseline subtraction method. However, the dynamic wave damage interaction between the incident guided wave and the damage causes constructive as well as deconstructive interference in the scattered waves and, with it, peaks and valleys in the WDIC patterns. Figure 8 shows a comparison of the numerical and experimental results of these WDIC patterns, i.e., the amplitude coefficients *C* and phase coefficients φ, for a constant damage thickness ratio τ=1 and varying damage orientation α. By the used collective representation, the position and shape of the peaks, i.e., high values, and valleys, i.e., very low values (dark blue color), can be observed for the WDIC patterns in dependency on the damage parameters τ and α.

In particular, the position and shape of the valleys, i.e., the very low *C* values in dark blue color, agree very well independently of the damage orientation α. Furthermore, the variations of both the amplitude coefficient *C* and phase coefficient φ patterns near the sensing angle $\gamma =0^{{}^{\circ}}$ with the damage orientation α are very low for the numerical as well as for the experimental results. This might be explained by the fact that this transmitted direction is shadowed by the damage independent from its orientation α. Generally, the numerical and experimental results show stronger deviation for the amplitude coefficients *C* in the transmitted direction, i.e., the sensing angle $\gamma =0^{{}^{\circ}}$. This might be explained by the influence of the adhesive layer between the damage and the plate in the experiment, which is not included in the presented numerical FE model. The influence of the adhesive layer on the numerical results is separately discussed in Section 5.4. Besides that, the amplitude coefficient *C* patterns match closely for both the amplitude values and the general shape.

At first glance, the numerical and experimental results for the phase coefficients φ seem quite different; see Figure 8i–p. This is to say that a phase of 0 (dark blue color) and 2π (yellow color) indicate the same phase and are only differentiated visually due to the selected representation. Hence, the significant jump in the phase coefficient φ patterns, which mostly align with valleys in the according amplitude coefficient *C* patterns, are basically smooth transitions. For example, the numerical and experimental phase coefficient φ results for a damage orientation $\alpha =45^{{}^{\circ}}$ and thickness ratio τ=1 in Figure 8l,p look very different from a purely visual point of view, although they differ only slightly.

However, the results for the experimental WDICs are evaluated using two excitation bursts with central frequencies $f_{c}=130\;\mathrm{kHz}$ and $f_{c}=200\;\mathrm{kHz}$ in successive measurements. Then, the WDICs are calculated for frequencies near these central frequencies to guarantee sufficient excitation; see Figure 7c. The excitation signals are selected to substantially overlap (see Figure 7c) and hence the usable fd range is increased significantly by combining their results. These experimental WDIC results are presented side-by-side, and their intersection at fd=152kHz can barely be seen due to the smooth transition between them; see Figure 8e–h. This indicates an adequate excitation of both excitation signals at this fd value. Consequently, the WDIC results from multiple time-domain measurements can be merged for carefully selected excitation signals.

In this study, the selected materials have solely isotropic behavior. Hence, the resulting WDIC patters for symmetric damages have to be symmetric as well, where the symmetry is measured according to the incident wave direction. This can be observed for the numerical results with damage orientations $\alpha =0^{{}^{\circ}}$ and $\alpha =45^{{}^{\circ}}$; see Figure 8a,i,d,l, respectively. An overall symmetry for the experimental results of these symmetric damages can be seen as well, but minor misplacement in terms of damage position and orientation or inconsistencies in the adhesive layer disturb the symmetry and cause visible deviations; see Figure 8e,m,h,p, respectively.

For the amplitude coefficients *C*, the back-scattered reflections show a dependency on the orientation α, basically following the law of reflections. Hence, the peak of the reflection rotates approximately at two times the angle of the orientation α; i.e., it is located near the sensing angle $\gamma =180^{{}^{\circ}}+2\alpha$. For example, the maximum of the reflected amplitudes for a damage orientation $\alpha =15^{{}^{\circ}}$ is close to a sensing angle $\gamma =210^{{}^{\circ}}$; see Figure 8b,f for the numerical and experimental results, respectively. Interestingly, this effect cannot be investigated for the phase coefficients φ, although there is a clear dependency on the damage orientation α. However, in contrast to the amplitude coefficients *C*, the values between the numerical and experimental results for the phase coefficients φ does not match as closely.

**Figure 9:**
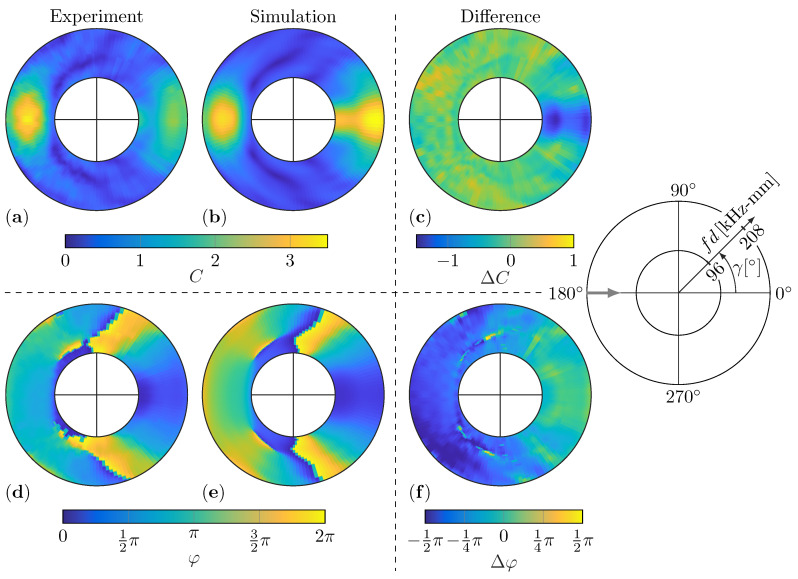
Comparison of numerical and experimental WDICs for τ=1 and orientation $\alpha =0^{{}^{\circ}}$: (**a**) experimental amplitude coefficients; (**b**) numerical amplitude coefficients; (**c**) difference of amplitude coefficients ((**a**) minus (**b**)); (**d**) experimental phase coefficients; (**e**) numerical phase coefficients; (**f**) difference of phase coefficients ((**d**) minus (**e**)).

For illustration of these differences in the amplitude coefficients *C* as well as the phase coefficients φ, the numerical and experimental results are interpolated and subtracted for a damage orientation $\alpha =0^{{}^{\circ}}$; see Figure 9. Therefore, the numerical and experimental WDICs are interpolated to a common grid every 2kHz-mm and $2.5^{{}^{\circ}}$.

The difference of the amplitude coefficients (see Figure 9c) show no structural differences except near the sensing angle $\gamma =0^{{}^{\circ}}$. This indicates a good overall agreement of the amplitudes and the valley positions. For the phase coefficients φ, the difference between the experimental (see Figure 9d) and numerical results (see Figure 9e) in Figure 9f reveals structural differences. Some of these might be caused by the necessary interpolation of the harsh jump before subtracting the results. However, in the right half of Figure 9f, i.e., sensing angles near $\gamma =0^{{}^{\circ}}$, the differences are rather small, whereby in the left half, i.e., sensing angles near $\gamma =180^{{}^{\circ}}$, the differences are relatively constant with $\Delta \varphi ≅-\frac{\pi }{2}$. These differences might be related to the missing adhesive layer in the numerical simulations, which is discussed in Section 5.4 in more detail.

**Figure 10:**
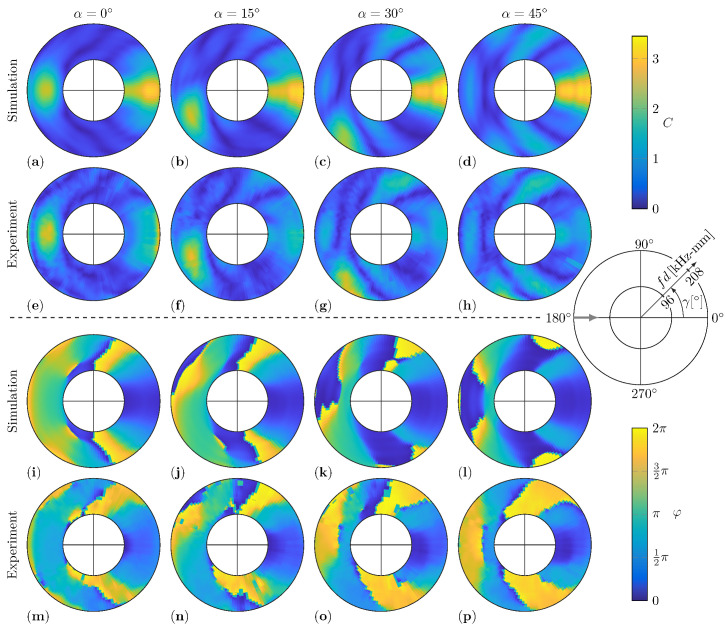
Comparison of WDIC results for τ=0.75 and different orientations α: (**a**–**d**) numerical amplitude coefficients; (**e**–**h**) experimental amplitude coefficients; (**i**–**l**) numerical phase coefficients; (**m**–**p**) experimental phase coefficients; (arrow in the coordinate system indicates incident wave direction).

**Figure 11:**
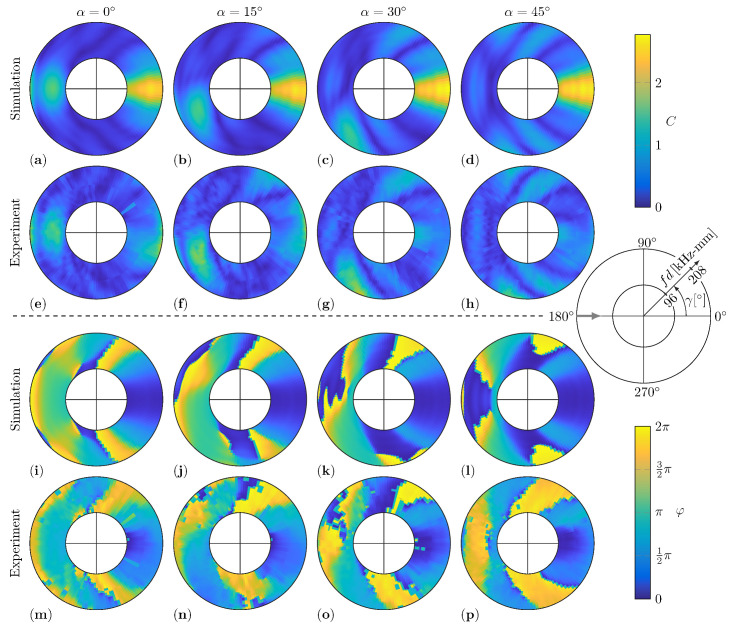
Comparison of WDIC results for τ=0.5 and different orientations α: (**a**–**d**) numerical amplitude coefficients; (**e**–**h**) experimental amplitude coefficients; (**i**–**l**) numerical phase coefficients; (**m**–**p**) experimental phase coefficients; (arrow in the coordinate system indicates incident wave direction).

Generally, for a decreasing thickness ratio τ, the values of the amplitude coefficients *C* are decreasing as well. This means that the amplitudes of the scattered waves are decreasing and the signal-to-noise ratio is becoming worse for the experimental measurements. Therefore, noise in the experimental results is more noticeable for thinner damages with τ=0.75; see Figure 10, and τ=0.5; see Figure 11. Besides that, not only is the amplitude level influenced by the thickness of the damage, but the shape of the WDIC patterns also changes with τ. For example, the peaks of the reflections in the amplitude coefficients *C* are decreasing and shifted to lower fd values; e.g., see Figure 8a, Figure 10a and Figure 11a for a sensing angle $\gamma =180^{{}^{\circ}}$ and damages with orientations $\alpha =0^{{}^{\circ}}$. However, the thickness ratio τ most does not affect the WDIC patterns as strongly as varying orientations α.

In addition to the comparison of the numerical and experimental WDICs of the different damage scenarios for the overall scattering pattern, the differences between them are presented by means of the root mean squared error (RMSE). The ${\mathrm{RMSE}}_{\Delta C}$ for the amplitude differences ΔC is defined as

(10) $$\begin{array}{c} {\mathrm{RMSE}}_{\Delta C}=\sqrt{\frac{1}{N}\sum_{fd,\gamma }{\left(C_{\mathrm{Exp}}-C_{\mathrm{Sim}}\right)}^{2}} \end{array}$$

and gives a scalar value for the differences between the experimental and the numerical WDIC patterns; see Figure 9c. Analogously, Equation (10) can be used to calculate the ${\mathrm{RMSE}}_{\Delta \varphi }$ for the phase differences Δφ by substituting the amplitude coefficients *C* by the phase coefficients φ to summarize the phase differences Δφ of the overall WDIC pattern; see Figure 9f. Therefore, the numerical and experimental WDICs are interpolated to a common grid (every 2kHz-mm and $2.5^{{}^{\circ}}$) containing N=8265 data points. Table 2 shows an overview of the ${\mathrm{RMSE}}_{\Delta C}$ and the ${\mathrm{RMSE}}_{\Delta \varphi }$ over all damage scenarios.

Generally, the errors in Table 2 have the same order of magnitude over all damage scenarios. It can be seen that the ${\mathrm{RMSE}}_{\Delta C}$ tend to increase slightly for increasing damage orientation α. Furthermore, the ${\mathrm{RMSE}}_{\Delta C}$ as well as ${\mathrm{RMSE}}_{\Delta \varphi }$ seem to decrease with the increasing thickness ratio τ. This might be explained by the fact, that for lower thickness ratios τ, the scattered guided wave signals are generally lower, and thus, the signal-to-noise ratio is decreased. The ${\mathrm{RMSE}}_{\Delta \varphi }$ are higher, but show similar behavior over the considered damage scenarios. However, this seems plausible due to the artificial errors (see Figure 9f) due to the interpolation of the jumps between 0 and 2π.

However, these characteristic WDICs may be used for damage identification by ultrasonic NDE or SHM by means of an array of integrated sensors, e.g., PWASs, and a comparison to a previously predicted WDIC database [31]. In such practical applications, amplitude and phase coefficients are measured and evaluated in the directions of fixed sensors; i.e., compared to the collective representation of the overall WDIC pattern used herein, much less data is available. Nevertheless, the variations of the WDICs over fd can be analyzed and utilized for damage identification [30,31]. However, for practical applications using PWAS arrays, certain issues are still to be clarified, e.g., how many sensors are necessary for a unique damage identification in the presence of measurement noise and other environmental influences. Such a PWAS measures a distributed value over its sensing area and influences the measured guided wave signal significantly in contrast to the contactless SLDV measurement of a single point. Additionally, the sensor signal might be a superposition of multiple guided wave modes, i.e., the sum of several WDIC patterns including mode conversion, which are difficult to separate with the limited number of measurement positions.

### 5.2. Influence of Baseline Subtraction

In the present paper, an improved baseline subtraction method is utilized to correct the positioning error of the plate when aligning the SLDV for the measurements of the top and the bottom sides of the pristine and damaged scenarios, respectively. As already explained in Section 4.2, the resulting time shift aligns these measurements and compensates the corresponding positioning error of the plate in the *x*-direction. The significant improvement of this correction for the baseline subtraction method is presented in Figure 5.

**Figure 12:**
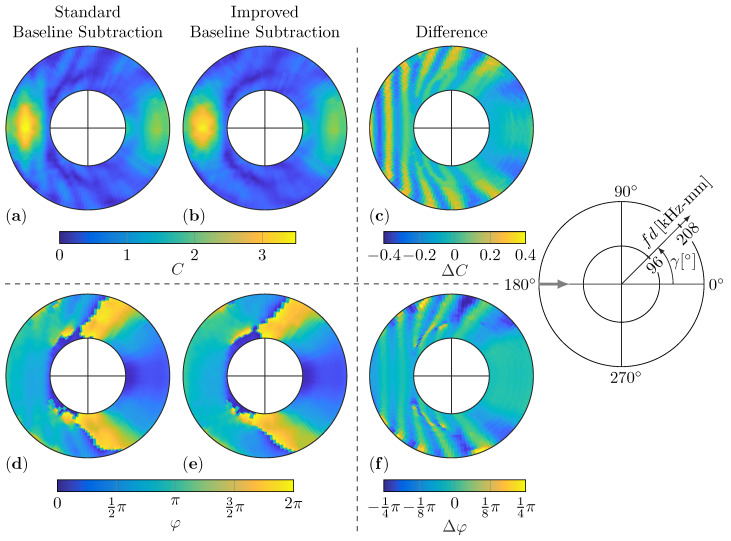
Experimental amplitude coefficients *C* and phase coefficients φ for a damage with τ=1 and orientation $\alpha =0^{{}^{\circ}}$: (**a**) amplitude coefficients *C* using baseline subtraction without correction; (**b**) amplitude coefficients *C* using advanced baseline subtraction; (**c**) amplitude coefficients difference ΔC ((**a**) minus (**b**)) reveals structural deviations caused by positioning error of the plate; (**d**) phase coefficients φ using baseline subtraction without correction; (**e**) phase coefficients φ using advanced baseline subtraction; (**f**) phase coefficients difference Δφ ((**d**) minus (**f**)) reveal structural deviations caused by positioning error of the plate.

However, Figure 12 illustrates the influence of the positioning error of the plate on the WDIC patterns.

Thus, the experimental WDICs are first calculated using the baseline subtraction without any correction; see Figure 12a,d. Second, the improved baseline subtraction to correct the plate misalignments in the *x*-direction is used for calculating the WDIC results; see Figure 12b,e. Significant differences can be noticed when comparing these results. Hence, the differences for the amplitude coefficients ΔC and the phase coefficients Δφ between these results are plotted in Figure 12c,f, respectively. It can be clearly seen that the patterns for both differences show similar structural differences. These differences are caused by misalignments of the plate of only approximately 0.1mm, which is the calculated alignment accuracy given by the SLDV for most cases. This corresponds to non-negligible systematic measurement errors of approximately 10% for the WDICs. This clearly demonstrates that the improved baseline subtraction not only affects the amplitudes of the scattered waves and, with it, the WDICs, but also minimizes the systematic errors causing structural differences in the WDIC patterns.

### 5.3. Dependency on Sensing Circle Diameter

**Figure 13:**
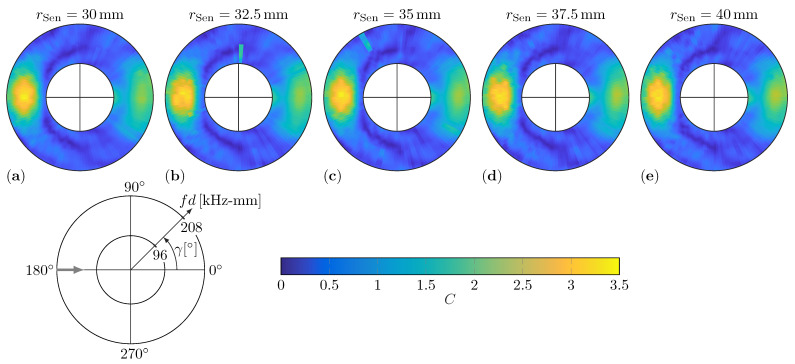
Experimental amplitude coefficient *C* results for damage orientation $\alpha =0^{{}^{\circ}}$ and thickness ratio τ=1 measured at different sensing radii: (**a**) $r_{\mathrm{Sen}}=30\;\mathrm{mm}$; (**b**) $r_{\mathrm{Sen}}=32.5\;\mathrm{mm}$; (**c**) $r_{\mathrm{Sen}}=35\;\mathrm{mm}$; (**d**) $r_{\mathrm{Sen}}=37.5\;\mathrm{mm}$; (**e**) $r_{\mathrm{Sen}}=40\;\mathrm{mm}$.

The CAFA idealizes the damage as a direction-dependent point source. However, in reality, the damage has a finite size. As already mentioned, all scattering phenomena during the wave damage interaction, e.g., scattering at the first and the second edge facing the incident waves, are represented by a single pair (*C* and φ) of WDIC patterns. Hence, the experimental WDIC patterns are examined for a possible dependency on the sensing circle size due to the finite size of the damage. In Figure 13, the experimental amplitude coefficients *C* for a damage orientation $\alpha =0^{{}^{\circ}}$ and thickness ratio τ=1 are compared for five sensing circle radii from $r_{\mathrm{Sen}}=30\;\mathrm{mm}$ to $r_{\mathrm{Sen}}=40\;\mathrm{mm}$ in steps of 2.5mm.

Besides the expected measurement noise, these results do not show noteworthy deviations. This is true for the amplitude values as well as for the general shape of the patterns, except for two clear outliers in Figure 13b,c. These can be explained by poor optical reflectivity of the respective measurement points, which causes high noise in the SLDV measurement signal. Generally, the surface treatment with the reflective spray results in an almost uniform reflectivity of the scan points along the sensing circles and such outliers are mainly avoided.

However, the amplitudes of the scattered waves are decreased with increasing sensing circle sizes due to wave attenuation of circular-crested waves. In other words, the signal-to-noise ratio deteriorates for larger sensing circles and measurement noise is more apparent in the WDIC patterns. However, the sensing circle has to be sufficiently large to only capture the propagating wave parts outside the near field of the complex wave damage interaction. Therefore, a sensing radius $r_{\mathrm{Sen}}=30\;\mathrm{mm}$ is sufficiently large to comply with the point-source-assumption of the CAFA, and all WDIC results in this study are evaluated and presented for this sensing circle size.

### 5.4. Influence of Adhesive Layer

**Figure 14:**
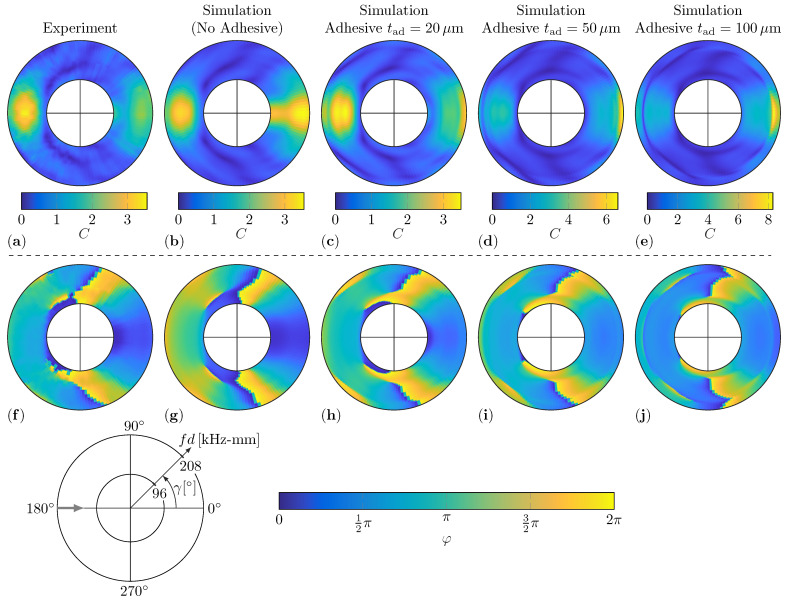
Comparison of different WDIC results for a thickness ratio τ=1 and an orientation alpha $\alpha =0^{{}^{\circ}}$: (**a**,**f**) experimental (sensing radius $r_{\mathrm{Sen}}=30\;\mathrm{mm}$); (**b**,**g**) numerical (without adhesive layer); (**c**,**h**) numerical with 20μm adhesive layer; (**d**,**i**) numerical with 50μm adhesive layer; (**e**,**j**) numerical with 100μm adhesive layer.

The comparison of the numerical and experimental WDIC results reveals certain discrepancies. In particular, the amplitude coefficients *C* in the transmitted direction near the sensing angle $\gamma =0^{{}^{\circ}}$ differ; see Figure 11a–h. These sensing angles are shadowed by the damage and the scattered guided waves have to pass the entire damaged area. Therefore, the differences near the transmitted direction might be related to the effect of the adhesive layer between the artificial damages and the host plate in the experimental setup. In the original numerical model, this bonding is idealized, i.e., the nodes on the bottom side of the damage and on the top side of the plate in the contact region are simply tied together. This may result in stronger scatter than the real adhesive bonding. To numerically investigate the influence of an adhesive layer, the FE model is adapted. A separate solid part is inserted between the damage and the plate representing the adhesive layer. This part is connected to the damage and the plate, again using tie connections for the nodes in the contact regions at the top and bottom side. Rectangular elements with an edge length of 0.5mm are used for the mesh of the adhesive layer, where a single element in the thickness direction is used. The material properties of the commercial superglue are modelled by an assumed Young’s modulus E=1.26GPa, Poisson’s ratio ν=0.3, and mass density $\rho =1070\;{\mathrm{kg}/m}^{3}$. Besides the material properties, the thickness of the adhesive layer $t_{\mathrm{ad}}$ mainly affects the results. Therefore, three scenarios with plausible thicknesses for superglue $t_{\mathrm{ad}}\in \left\{20\;\mu m,50\;\mu m,100\;\mu m\right\}$ are simulated to study its influence. These simulation results with the adhesive layer are compared to the original numerical (without adhesive layer) and experimental ones in Figure 14.

The adhesive layer added in the numerical simulation softens the connection between the damage and the plate due to its lower stiffness and, with it, clearly affects the WDIC patterns. In general, differences are more significant for thicker adhesive layers. The amplitude coefficients *C* near the transmitted direction $\gamma =0^{{}^{\circ}}$ are highly affected by the adhesive layer; e.g., see the simulation results with a $t_{\mathrm{ad}}=20\;\mu m$ thick adhesive layer in Figure 14c in comparison to the ones without an adhesive layer in Figure 14b. This might be explained by the fact that the transmitted waves are passing through the entire damage area influenced by the adhesive layer. Hence, the discrepancies between the experimental results and the simulation results can be reduced in this transmitted direction ($\gamma =0^{{}^{\circ}}$) by adding an adhesive layer in the simulation; compare Figure 14a,c. However, the shape and position of the valleys in the amplitude coefficients *C* patterns change as well and tend to be shifted to lower fd-values for increasing thickness of the adhesive layer. This can be observed in the left half of the scattering patterns, i.e., between $\gamma =90^{{}^{\circ}}$ and $\gamma =270^{{}^{\circ}}$, where an additional valley is slowly entering the visualized fd-range for increasing thicknesses; see Figure 14a–e.

The influence of the adhesive layer in the simulation can be seen in the phase coefficients φ patterns as well; see Figure 14f–j. The overall values for the phase coefficient φ patterns for the simulation with an adhesive layer of $t_{\mathrm{ad}}=20\;\mu \mathrm{mm}$ thickness resembles the experimental results (see Figure 14f) more closely than the simulation without adhesive; see Figure 14g. Interestingly, the adhesive layer influences the transmitted and reflected directions in opposite directions; i.e., the phase coefficients φ are increased ($\gamma =0^{{}^{\circ}}$) and reduced ($\gamma =180^{{}^{\circ}}$), respectively. However, the smallest adhesive layer thickness $t_{\mathrm{ad}}=20\;\mu m$ is already a very thin adhesive film and the real one in the experiments might be actually thicker. Hence, the assumed stiffness of the adhesive layer material might to be too low. However, this parameter study for the thickness of the adhesive layer already clearly shows its influence. It seems to be promising that the discrepancies between the experimental and simulated WDIC patterns can be further reduced by introducing a properly modeled adhesive layer. Furthermore, a comprehensive parameter study varying the thickness as well as additional parameters would be necessary to examine the influence of each parameter separately and to optimally fit the numerical WDIC results to the experimental ones.

## 6. Conclusions

This study comprehensively investigates both amplitude and phase WDIC patterns for the interaction between guided waves and structural discontinuities, e.g., damages, for numerical simulation and experimental measurement results in detail. The utilized numerical FE model is specially designed to simulate these WDICs directly in the frequency domain. Furthermore, the compact size of this FE model enables numerically efficient WDIC predictions. For the experimental setup, the guided waves in a thin aluminum plate are excited by piezoelectric transducers. The incident and scattered guided wave fields are measured by a single SLDV. Squared miniature steel sheets are bonded to the top surface of the host plate as local changes in the structure to imitate damages with different characteristics, i.e., orientation and thickness. These damage characteristics are described by dimensionless parameters based on similitude theory. The presented WDIC results can therefore be used universally for any geometrically scaled scenario. Generally, WDICs are highly sensible damage features, i.e., they depend solely on the damage characteristics for a given host structure, and their resulting scattering pattern is uniquely described. These scattering patterns are visualized by a novel collective representation that enables holistic observation of dependencies on the damage characteristics. A detailed comparison between numerical and experimental results for complex-valued WDICs, i.e., amplitude and phase coefficients, is discussed. In particular, the comprehensive investigations of the phase coefficients are valuable and provide additional information for possible WDIC-based ultrasonic NDE or SHM applications. However, the WDIC scattering patterns reveal a clear dependency of the peaks in the back-scattered reflections for both the numerical and experimental amplitude coefficient on the damage orientation, basically following the law of reflection. Generally, numerical and experimental results show good agreement. Discrepancies between numerically and experimentally found WDIC patterns for the phase and the amplitude in the transmitted direction can be mainly assigned to the influence of the adhesive layer between the damage and the plate. Therefore, the numerical results of an adapted numerical model including an adhesive layer with varying thicknesses are investigated to show its high influence on the WDIC patterns. Generally, this paper attempts to guide researchers and practitioners in the investigation of WDICs. Therefore, challenges in the experimental measurements are highlighted and possible solutions, e.g., an improved baseline subtraction method, are proposed and discussed. This proposed improved baseline subtraction method compensates for the misalignment of the guided wave signals in the experiments, e.g., for the same scan point position in the pristine and damaged case, due the positioning error of the plate. In general, this correction in the baseline subtraction method might be useful for researchers and practitioners in ultrasonic NDE applications using contactless measurement systems, such as an SLDV. In this paper, the improvement of the baseline subtraction is demonstrated and its effect on the WDIC patterns is presented, thereby clearly showing structural differences when neglecting this positioning error of the plate, i.e., using the standard baseline subtraction without compensation. Furthermore, the sensitivity of WDICs for selected sensing radii is studied experimentally, showing only a minor influence mainly related to measurement noise, thus confirming the point-source-assumption required for the application of local-global approaches such as the CAFA method.

Future research will investigate possible applications of WDICs in advanced damage identification methods. Therefore, several issues, e.g., the number of sensors required for an unique damage identification or the superposition of multiple guided wave modes due to mode conversion in a single sensor signal, are still to be clarified. Furthermore, the limited database of simulated WDICs could be substantially extended by machine learning algorithms, e.g., deep neural networks [31]. The experimental WDIC results from the presented study could improve the prediction accuracy for previously unseen damages by machine learning algorithms trained on simulation data, which is known as transfer learning. These transfer learning methods require only a limited number of generally costly and time intensive experiments, and seem promising for future research. Moreover, WDIC predictions by numerical simulation and experimental measurements for real damages, e.g., fatigue cracks or delaminations, should be addressed to enable applications in ultrasonic NDE and SHM.

## Figures and Tables

**Table 1:** Material properties.

Parameter	Aluminum	Steel
Young’s modulus *E*	70 GPa	210 GPa
Poisson ratio ν	0.33	0.3
Mass density ρ	$2700\;{\mathrm{kg}/m}^{3}$	$7800\;{\mathrm{kg}/m}^{3}$

**Table 2:** ${\mathrm{RMSE}}_{\Delta C}$ and ${\mathrm{RMSE}}_{\Delta \varphi }$ for all damage scenarios.

		${\mathrm{RMSE}}_{\Delta C}$				${\mathrm{RMSE}}_{\Delta \varphi }$			
		α				α			
		$0^{{}^{\circ}}$	$15^{{}^{\circ}}$	$30^{{}^{\circ}}$	$45^{{}^{\circ}}$	$0^{{}^{\circ}}$	$15^{{}^{\circ}}$	$30^{{}^{\circ}}$	$45^{{}^{\circ}}$
τ	0.5	0.443	0.473	0.509	0.552	0.969	1.229	0.751	1.051
	0.75	0.431	0.434	0.534	0.560	0.981	0.847	0.959	1.046
	1	0.365	0.412	0.462	0.366	0.855	0.745	0.632	0.679

## Data Availability

Not applicable.

## Abbreviations

The following abbreviations are used in this manuscript:

CAFA	Combined Analytical Finite Element Model Approach
FE	Finite Element
NDE	Non-Destructive Evaluation
NRB	Non-Reflective Boundaries
PWAS	Piezoelectric Wafer Active Sensor
SHM	Structural Health Monitoring
SLDV	Scanning Laser Doppler Vibrometer
SSD	Steady-State Dynamics
WDIC	Wave Damage Interaction Coefficient
